# Application of hiPSC as a Drug Tester *Via* Mimicking a Personalized Mini Heart

**DOI:** 10.3389/fgene.2022.891159

**Published:** 2022-04-14

**Authors:** Li Wei, Shutao Xia, Yifei Li, Yan Qi, Yue Wang, Donghui Zhang, Yimin Hua, Shuhua Luo

**Affiliations:** ^1^ Key Laboratory of Birth Defects and Related Diseases of Women and Children of MOE, Department of Pediatrics, West China Second University Hospital, Sichuan University, Chengdu, China; ^2^ State Key Laboratory of Biocatalysis and Enzyme Engineering, School of Life Science, Hubei University, Wuhan, China; ^3^ Department of Cardiovascular Surgery, Pediatric Heart Center, West China Hospital, Sichuan University, Chengdu, China

**Keywords:** IPSC, disease model, tissue engeneering, heart in dish, drug testing

## Abstract

Human induced pluripotent stem cells (hIPSC) have been used to produce almost all types of human cells currently, which makes them into several potential applications with replicated patient-specific genotype. Furthermore, hIPSC derived cardiomyocytes assembled engineering heart tissue can be established to achieve multiple functional evaluations by tissue engineering technology. This short review summarized the current advanced applications based on the hIPSC derived heart tissue in molecular mechanisms elucidating and high throughput drug screening.

## Introduction

Human-induced pluripotent stem cells (hIPSC) are currently recognized as the most suitable cell source for producing almost all types of human cells. The interest in reprogramming cell fate has led to a revolutionary change in regenerative medicine, which achieves a “copy-paste” of patient-specific genotype cells from the bench to the bedside (Carvajal-Vergara et al., 2010; Itzhaki et al., 2011; Yazawa et al., 2011; Kim et al., 2013). In the past decade, researchers in the field of hIPSC have attempted to solve a series of fundamental issues, including whether hIPSC have the ability to differentiate into different types of functional cells (Ramalho-Santos, 2009; Maher, 2013; Zhao et al., 2019; Cowan et al., 2020; Lee et al., 2022); whether hIPSC-derived cells could mimic pathophysiological processes; and whether this completely “*in vitro*” model could help researchers unravel underlying pathogenetic mechanisms and provide clues on diagnostic and therapeutic choices (Pavez-Giani and Cyganek, 2021; Faulkner-Jones et al., 2022; Yang et al., 2022). Along with their performance in several functional tests, hIPSC-derived cells show unique advantages, although even fully mature tissue characteristics still cannot be achieved today. As more complicated syndromes have been modeled, hIPSC-based disease modeling has been praised as a simple tool to obtain essential knowledge of complicated molecular pathogenetic processes (Park et al., 2019; Liu et al., 2021). Undoubtedly, high-throughput drug testing for particular diseases based on engineering of heart tissue (EHT) using hIPSC has given birth to the transfer of *in vitro* models to more productive applications (Lam et al., 2019).

## Limitations to Drug Development

Investment in drug development is considered a high-risk enterprise and requires an extremely prolonged commitment. Irrespective of the chemical compounds, small peptides, microRNAs, or even gene delivery approach, drug screening methods need to be complemented by various substrates once the candidate libraries have been built (Liu et al., 2022). Traditionally, the toxicity of new drugs has been tested using different types of cell lines. Regardless of cost and efficiency, a simple cell line test supplies information to allow the selection of preliminary candidates and confirms an active serum concentration. *In vitro* drug screening then must advance into physiological experiments using animal models. Mice have been involved for the initial process. And transgenic mice are critical for inherit cardiovascular diseases. Then large animals are considered as the further step before clinical transition to measure specific cardiac electrophysiology. This approach can lead to the simulation of potential drug reactions in different organs throughout the body, although numerous failures have reminded researchers that interspecies differences may represent a barrier to fully transfer experimental test results to clinic practice (Pavez-Giani and Cyganek, 2021; Bourque et al., 2022; Tricot et al., 2022). Then the human based clinical trial is critical to fully validate a newly invented drugs. Commonly, this process would take more than 10 years. However, due to various genetic and epigenetic backgrounds between animal models and human, even within humans, differences in sex hormone-cardiac-electrophysiology, races, environmental exposure, failure always been obtained for final pharmacy validations leading to huge waste of costs and time. As in the Long-QT syndrome, genetic ablation of *KCNQ1* in mice did not consistently lead to a cardiac phenotype similar to that of the observed in the long-QT syndrome in patients, which is due to differences in the functions of potassium channels between humans and mice; thus, it is not scientifically feasible to perform cardiotoxicity testing of drugs as there is a risk of inducing prolongation of the QT interval (Casimiro et al., 2001). Accordingly, there is an obvious gap in translational medicine research, which has raised the urgent demand for a humanized functional test model. In this context, hIPSC-derived human functional cells may perfectly fit this purpose. The marked increased in differentiation efficiency exceeds the limits placed on cell numbers (Klug et al., 1996), and the completely *in vitro* culture and maturation models provide the opportunity to build a heart organoid. Moreover, EHT (Kehat et al., 2001; Paik et al., 2020) and even a three-dimensional (3D) ventricle (Liang and Zhang, 2013) have been proposed to mimic cardiac tissue and heart physiological functions at various levels.

## Human-Induced Pluripotent Stem Cells is a Natural Choice for Cell Modeling

Early in 1996, human beating cells were first observed following spontaneous Emory body (EB) differentiation (Klug et al., 1996). Unfortunately, the differentiation efficiency was really low, which was around 5–10%. Subsequently, more than 20 years were spent improving cardiomyocyte differentiation protocols. Since Yamanaka’s research team reported the convinced method to improve the production of hIPSC, several studies devoted to establish a stable protocol to simulate the differential efficiency. Particular regulation of several signaling pathways, which were involved in cardiomyocytes development including bone morphogenetic protein (BMP), wingless/integrated (Wnt) and transforming growth factor-β (TGF- β), had been indicated to promote efficacy of hIPSC derived cardiomyocytes (hIPSC-CMs), which could reach more than 90% purity of CMs. Currently, cardiomyocytes differentiation efficiency is now one of the most robust among all the differentiation lineages. Although, the evaluation and qualification of hIPSC-CMs did not achieve cell cluster contractility, it can be applied to cell-based assays, including differentiation efficiency, cardiomyocytes structure (Kehat et al., 2001), single cell contraction, and electrophysiology. The development of an efficient, stable, and low-cost differentiation model stimulated an unprecedented boost in subsequent research activity. However, it is important to be noted that the hIPSC-CMs demonstrated phenotypic plasticity. And current differential protocol could only derived CMs into neonatal or under-maturated stages, which could not be fully identified as matured CMs. So that, the cultural conditions and duration are critical to make hIPSC-CMs function. Thus, it should be build a specific protocol for differentiation according to genotypes and applications of hIPSC-CMs. Specific gene expressions had been used to determine which stages the hIPSC-CMs remaining, such as mesoderm formation (*MIXL1* and *BRY*), cardiogenic mesoderm (*ISL1* and *MESP1*), cardiac-specific progenitors (*GATA4*, *TBX5* and *NKX2.5*) and relative mature CMs (*TNNT2* and *MYH7*). Besides, ventricular and atrial specific differentiation could be achieved recently, which provided more platforms to assess the drugs targeting different cellular types respectively. A series of studies have evaluated whether the methods could be designed to improve cardiomyocyte maturation *in vitro* (both at the cell level and at the micro-tissue level). EHT constructs greatly expanded the potential and breadth of functional testing, presenting an *in vitro* functional assay a model with a phenotype that may truly mimic clinical symptoms. Successful cardiomyocyte modeling shifted the hIPSC-CM-based drug screening model from a traditional target driven assay to a functional model, which expands the strategies for drug validation and measurement of pharmacodynamic effects (Paik et al., 2020). So that, if EHT had been used to test the adverse effects on cardiac development, it should to make the hIPSC-CMs remained in specific developmental phases. While exploring the therapeutic effects on mature CMs, purification and advanced protocol should be applied to make EHT functional contraction and electrical activity. As shown in a recent study, four types of calcium channel blockers initially showed therapeutic efficacy, but the mechanism of action and transcriptional activity differed in hIPSC models, and partly explained the origins of drug resistance and unexpected side effects (Lam et al., 2019).

## Mimicking Disease Phenotype of Human-Induced Pluripotent Stem Cells-Derived *in vitro* Tissues

One of the most well-known advantages of hIPSC is the generation of personalized genotype cells. Although more than 99.9% of the genome sequence is exactly the same across populations, only a 0.1% difference is responsible for the diversity in the response to different drugs. From this standpoint, hIPSC-based drug testing will represent a simple and effective model to verify personalized-based outcomes. So that, to establish a personal genotype based EHT and determine the best drug applications according to a specific genetic background could be considered as the personalization strategy, which would reduce the differences between population results and individual application. In a previous study (Lam et al., 2019), based on only three different cell lines, four drugs showed different response combinations. Although we could explain the variability in gene transcription in cell lines due to genetic variants and not to drugs, another explanation is also due to the variability of differently expressed differentiation characteristics within the same genotype (Liang and Zhang, 2013). Accordingly, another important question has been raised: is there a standard mechanism involved in cardiac differentiation and EHT assembly? Since 2001, cardiomyocyte differentiation has undergone extensive research to identify how to produce mature cells. From the “beating” phenotype to the detailed subcellular structure (Chung et al., 2007; Qi et al., 2022), even atrial or ventricular cardiomyocytes exhibit a specific physiology, which has gradually become a new standard in the field (Kattman et al., 2011). At this point, the development of universal quality standards for EHT will promote the scientific advancement among different research groups and will direct the development of specifically targeted drugs. In addition, Zhang et al. (2013). identified an engineered tissue patch function was associated with cardiomyocyte purity within the tissue. Interestingly, contractility showed the highest score in 65–75% of cardiomyocytes and the conduction velocity was positively associated with purity. This finding suggests that it is possible to establish a specific functionally-enhanced tissue model not only via a spatial organization pattern but also by establishing a subtype ratio.

The specific functionally enhanced tissue model could be used directly to model a target disease dysfunction, or different functional characteristics of the same disease. Most importantly, the mimicking mini hearts share the same genetic background, which could validate novel therapeutic strategies. Beyond the applications of drug tests on various genetic backgrounds, hIPSC-CM-EHTs have been used for gene mutation induced inherit cardiovascular diseases. Over the last decade, several cell lines of genetically inherited diseases have been developed to test the efficacy of old and newly invented drugs, including *LMNA* (Lee et al., 2019), *TTN* (Hinson et al., 2015), *MYH7* (Vander Roest et al., 2021), *TNNT2* (Pettinato et al., 2020), *MYBPC3* (Seeger et al., 2019), *PKP2* (Kim et al., 2013), *DSP* (Xia et al., 2020), *TAFAZZIN* (Wang et al., 2014)*, PTPN11* (Carvajal-Vergara et al., 2010)*-*induced cardiomyopathy, and *RYR2* (Park et al., 2019), and *KCNQ1* (Casimiro et al., 2001) induced arrhythmia. Furthermore, multiple genetic variants have also been evaluated using iPSC-based models, which has provided a further understanding on the complex genetic background (Gifford et al., 2019). Thus, the *in vitro* heart may represent an optimal approach for cardiac research both in terms of the mechanisms of inherited cardiovascular disease and screening of new drugs.

## Application of the Human-Induced Pluripotent Stem Cells-Derived Cardiomyocyte-Assembled Engineered Heart Tissue

Accordingly, we could easy hypothesize that the application of the hIPSC-derived cardiomyocyte-assembled EHT (hIPSC-CM-EHT) model will define a novel drug screening model system. In this system, the variable genetic background of the micro-tissue will play the role of a tiny drug tester, for different genotypes, thus, different types of functional micro-tissue models can be used to mimic different clinical phenotypes. According to current strategies, there are two kinds of common aspects of EHT as 3D architecture and monolayer. Usually, monolayer model is more suitable for high throughput screening. And this kind of monolayer model is great for evaluation for toxicity, proliferation, development, and even for calcium transit. While 3D structural model is more difficult to be established and reduce the capability for large size screening. But it presents the advantages in determining contractile activity and interaction between various cells and extracellular matrix. Thus, to select the optimal model based on the propose of research is also crucial for drug screening. Due to the different strategies to build EHT, each type of EHTs presents different capabilities in determining contractile parameters (Table 1). Generally. There are five approved strategies of EHT establishment. Organoid has been used to mimic the function of whole heart with several approaches (Lewis-Israeli et al., 2021). Although it could be recorded contraction, but it still doesn’t fully address the cardiac function with very limited parameters could be underlined. Then thin film has been widely used to measure part of contractile activities (Wang et al., 2014). But it could not be loaded mechanical stress and still a kind of 2D EHT. EHTs based on muscle ring/network/bundle/biowire satisfied most of contractile parameters measurements (Zhang et al., 2013; Zhao et al., 2019; Li et al., 2020; Bliley et al., 2021). However such models required more hIPSC-CMs and complex steps, which would reduce the capability of high throughput. Similarly, *in vitro* cardiomyocyte maturation methods, for example, electrostimulation (Sun and Nunes, 2016; Ronaldson-Bouchard et al., 2018) and mechanical rocking (Zhang et al., 2013; Shadrin et al., 2017), will represent additional options to mimic a differentiated adult-like phenotype of cardiac tissue function. Although the engineered “maturation” of cardiomyocytes has always been difficult, it is also difficult to derive equivalent EHT at a specific stage of development. Nonetheless, the hIPSC-CM-EHT model is an easy-to-harvest artificial heart tissue, compared with *in vivo* human heart tissue specimens, which presents similar functional properties (Eschenhagen et al., 2015).

**TABLE 1:** The application of various types of EHTs.

Assessment parameters	Biological meaning	Organoid	Thin firm	Muscle ring	Muscle network	Muscle bundle/biowire
Stress loading				+	+	+
Passive force				+	+	+
Inotropic response to compound	Receptor sensitivity	a+	+	+	+	+
Excitation threshold		+	+	+	+	+
Post-Rest Potentiation		+	+	+	+	+
Active force	Contractile capability	Calculating result	Calculating result	+	+	+
Frank-Starling relationship				+	+	+
Maximum contraction rate		+	+	+	+	+
Force-frequency relationship		Calculating result	Calculating result	+	+	+
Measuring method		Video	Video	Video/Sensor	Sensor	Video/Sensor

+, indicates the actual measurement results.

**FIGURE 1:**
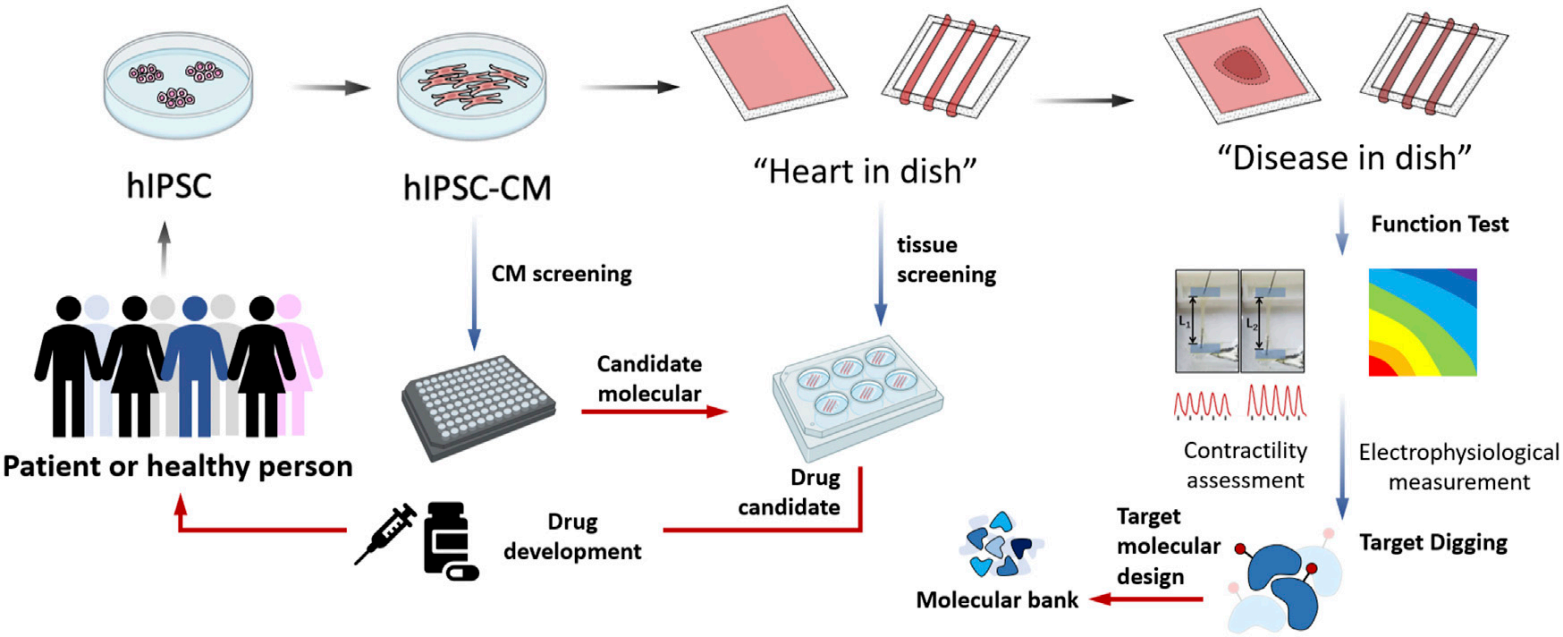
The applications of hIPSC derived cardiomyocytes assembled engineering heart tissue model in cardiovascular researches.

Considering the advantages of such a model system, it may be possible to mimic the chronic side effects of prolonged treatment (years) with specific agents only after a few weeks and rare phenotypes may be rapidly identified *in vitro*. Furthermore, the process of cardiomyocyte differentiation and cardiac tissue assembly provides an extended treatment window able to mimic almost all periods of heart development. A recent study by Theodoris et al. (2021) established a network-based screening strategy using iPSC-derived cells to identify therapeutic candidates for heart valve disease, which was made feasible by human iPSC technology, network analysis, and machine learning and represents an effective path for drug discovery. In particular, due to the limits posed by ethical issues, this model system provides the valuable opportunity to focus on the “leopard spot” transformation of cardiomyocytes specificity, proliferation, and maturation directly form the fetal cell’s black box. So that, the application of hIPSC-CM-EHT could help to accelerate the drug screening by the following steps (Figure 1). First, hIPSC should be obtained from a specific group or individual patients and healthy population. Then the basic hIPSC-CM could take over the initial candidate molecules screening. After purification of CMs, “heart in dish” could be established and involved in tissue screening, and benefit drug development, impacting the therapeutic strategy for the donors representing population. Then, specific genotype hIPSC-CMs would be participated in building “disease in disease”, which had been used in functional measurement, providing information for target digging and molecular design.

During the worldwide outbreak of COVID-19, the hIPSC-CM-EHT model has contributed to several types of investigations that: 1) mimicked myocardial injuries caused by SARS-CoV-2 infection (Li et al., 2021; Perez-Bermejo et al., 2021; Salerno et al., 2021; Xie et al., 2022); 2) evaluated the safety of different types of vaccines including mRNA and adenovirus vaccines, especially across different ethnic populations with different genomic backgrounds (Bojkova et al., 2020; Lu et al., 2020; Davidson et al., 2021); and 3) assessed the potential side effects of anti-COVID-19 developed treatments on cardiomyocytes (Charrez et al., 2021; Deguchi et al., 2021; Dimai et al., 2021). Research involving the hIPSC-CM-EHT model in COVID-19 has rapidly extended the application of hIPSC, making it a more common conceptual and powerful investigative tool.

The application of hIPSC-CM-EHT as a systematic, standardized, and high throughput tool will be the objective next step, and much more research will stem from here.
