# Supplementary material for: Application of hiPSC as a Drug Tester Via Mimicking a Personalized Mini Heart
Source: Front Genet. 2022 Apr 14;13:891159. doi: 10.3389/fgene.2022.891159 (PMC9046785; doi:10.3389/fgene.2022.891159)
Supplement: Supplementary file 1 [file Table1.DOCX]

**Table 1. The application of various types of EHTs**

| Assessment parameters | Biological meaning | Organoid | Thin firm | Muscle ring | Muscle network | Muscle bundle/biowire |
| --- | --- | --- | --- | --- | --- | --- |
| Stress loading |  |  |  | + | + | + |
| Passive force |  |  |  | + | + | + |
| Inotropic response to compound | Receptor sensitivity | + | + | + | + | + |
| Excitation threshold |  | + | + | + | + | + |
| Post-Rest Potentiation |  | + | + | + | + | + |
| Active force | Contractile capability | Calculating result | Calculating result | + | + | + |
| Frank-Starling relationship |  |  |  | + | + | + |
| Maximum contraction rate |  | + | + | + | + | + |
| Force-frequency relationship |  | Calculating result | Calculating result | + | + | + |
| Measuring method |  | Video | Video | Video/Sensor | Sensor | Video/Sensor |

+, indicates the actual measurement results.
